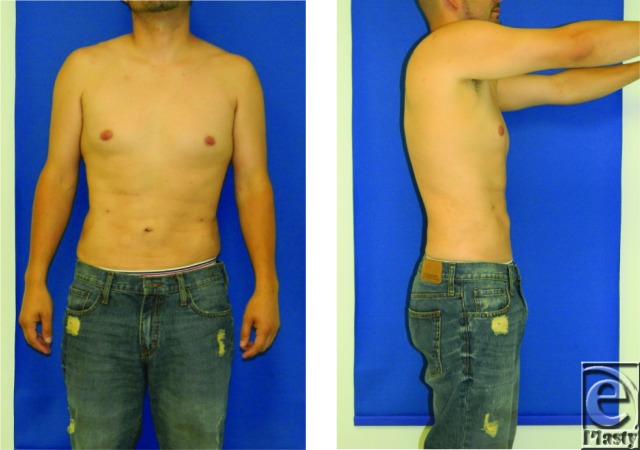# Liposuction

**Published:** 2013-01-15

**Authors:** Sachin M. Shridharani, Howard D. Wang, Navin K. Singh

**Affiliations:** Department of Plastic and Reconstructive Surgery, The Johns Hopkins University School of Medicine, Baltimore, Md

**Figure:**
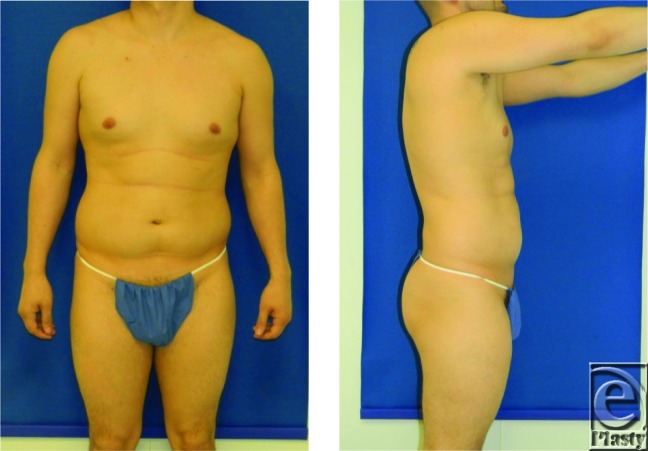


## DESCRIPTION

A 36-year-old man presents to the plastic surgeon's office for consultation regarding his desire for liposuction. He is overall healthy with a nonsignificant medical history. His height and weight are 166 cm and 68.2 kg, respectively.

## QUESTIONS

1. **Identify the location of lipodystrophy in this patient?**
2. **What is the recommendation regarding large-volume liposuction?**
3. **What are the different types of wetting solutions?**
4. **What is the maximum safe dose of lidocaine used within the wetting solution for this patient?**
5. **What measures should be taken to reduce the risk of deep venous thrombosis (DVT) and pulmonary embolism (PE) in patients undergoing liposuction?**
6. **Describe the postoperative management of this patient after liposuction?**

## DISCUSSION

Lipodystrophy can present in various areas of the body, and subcutaneous fat is amenable to improvement with liposuction. Concepts regarding the management of lipodystrophy have advanced from simply “suction” to lipo “sculpture.” The most commonly treated areas are abdomen, flanks, thighs, chin, knees, back, buttocks, and breast. Male patients tend to treat flanks, lower abdomen, and chest (for gynecomastia and/or pseudogynecomastia). This patient, with a body mass index (weight in kg / (height in m^2^) of 24.75, has excessive fat deposition in his abdominal and flank regions, and he seeks body contouring in these specific areas. Liposuction is a very effective procedure employed to treat localized adipose deposits. Many techniques are available: suction-assisted, ultrasound-assisted, power-assisted, laser-assisted, and power water-assisted liposuction. The choice of technique often depends on patient characteristics and surgeon preference. In patients with excess abdominal fat seeking liposuction, careful physical examination of the region is important to assess for the presence of incisional hernias from prior surgery, umbilical hernias, and whether the excess fat is extra- or intra-abdominal in origin.^1^

During preoperative consultations with the patient, patient expectations in terms of the goal of liposuction as contouring as opposed to weight reduction have to be clear. The timeline for healing over several weeks is shared with the patient. Detailed discussion of risks and benefits should be undertaken including the logistics, nature of the procedure, pain management, expected time course of recovery, expected course of management of complications, and warning signs and symptoms of postoperative complications. The patient should be shown representative before/after pictures of patients with similar body habitus as himself or herself. Alternative methods of management should be discussed alongside the best, average, and worst case scenarios related to the proposed surgery. The patient should understand the risks/benefits and warning signs/symptoms of side-effects of medications and treatments to be used in the procedure. In communicating expected amounts of fat removal, some physicians use units such as cubic centimeter, grams, or pounds. To facilitate understanding of medical volumes for patients who are not accustomed to conceptualizing volumes in terms of metric quantities, common volume measurements of everyday items such as soda-cans (355 cc or 12 oz) or wine bottles (750 cc) may be utilized. Others may describe number of inches to be removed around a waistline or thigh line. Regardless of the method used, it is important to have documentation of preoperative weight and linear measurements for appropriate comparison after the operation, in addition to standard medical photography.

Once the decision to proceed with liposuction is made, the appropriate setting to perform the operation should be decided. The overwhelming majority of liposuction cases can be done on an outpatient basis. In some circumstances, based on individual patient variables, patients may either prefer to be admitted overnight to a hospital or a “Short-stay 23 hours” facility, or the surgeon may find that the patient is not suitable to be discharged home and have to be admitted. The estimated total volume of aspirate to be removed impacts this decision as well. The American Society of Plastic Surgeons recommends that large-volume liposuction, which is defined as total aspirate volume greater than 5000 mL, should be performed at an acute care hospital, where postoperative vital signs and urinary output may be monitored overnight.^2^

One factor that has made large-volume liposuction significantly safer is the advancements in wetting solutions. At the advent of liposuction, the dry technique was utilized. No fluid is infiltrated into the subcutaneous tissue with the dry technique, and the suction aspirate consists of 20% to 45% blood. As it became more obvious that this level of patient blood loss was unacceptable, the wet technique, which entails injection of 200 to 300 mL of wetting solutions into each area, came into use. This lead to a decreased volume of blood to 4% to 30% of the aspirate volume. The superwet technique uses 1 mL of wetting solution, which includes epinephrine and sometimes lidocaine, for each milliliter of fat to be aspirated. A significant reduction in blood loss is associated with this technique. Blood loss of less than 1% of aspirate volume can be achieved. Finally, the tumescent technique involves the largest volume of infiltrate. Three to 4 mL of solution is injected for each milliliter of fat planned to be aspirated. Estimated blood loss is less than 1% of aspirate.^3^ This patient received a total of 3 L of wetting solution. The first 2 L contained 50 mL of 2% lidocaine, 1 mL of 1:1000 epinephrine, and 20 mL of bicarbonate, and a third liter contained 25 mL of 2% lidocaine instead of 50 mL. His total aspirated volume was 2.4 L.

While the use of tumescent technique and improved understanding of fluid management have made liposuction a safer procedure, serious complications can still occur. The most common sequelae after liposuction is contour deformity. Other serious complications include infection, bleeding, pain, seroma, DVT, PE, fat embolism, skin necrosis, vital organ perforations, and adverse events associated with anesthesia.

Several types of anesthesia are used for liposuction. There is no ideal method as there are pros and cons to each, and the choice depends on the patient's overall health, estimated volume of aspirate to be removed, and individual patient preference. Some patients may prefer local-only anesthesia or intravenous sedation or even general anesthesia on a case-by-case basis. Since patient expectations often center on minimizing down-time by selecting a minimally invasive procedure, most liposuction can be done awake, especially when it is facilitated with laser liposuction. The most common anesthetic agent included in wetting solutions is lidocaine. High levels are associated with toxicity manifested as cardiac and neurologic complications. Less than 7 mg/kg of lidocaine is usually recommended; however, a higher dose is safe when lidocaine is added to liposuction wetting solution because of several favorable factors. These factors include a slower absorption of lidocaine in fat since lidocaine is lipophilic, decreased absorption due to vasoconstriction induced by epinephrine, and the removal of lidocaine via the liposuction aspirate. Some of the lidocaine infiltrate also continues to ooze out of the liposuction portals over the first 24 to 48 hours. When used with epinephrine in wetting solution, lidocaine dosage of 35 mg/kg is generally accepted as safe.^4^ Higher dosages of lidocaine up to 55 mg/kg have also been reported to be used safely.^5^ Our patient weighs 68.2 kg, and thus his calculated maximum safe dose of lidocaine based on a safe dosage of 35 mg/kg is 2387 mg.

Another major complication of liposuction is DVT/PE. Preoperative evaluation of patient's risk factors for thrombosis helps to guide the implementation of prophylactic measures. Measure such as compression stockings, pneumatic compression device, and anticoagulation therapy should be considered. While less common than pulmonary emboli, fat emboli is another reported complication of liposuction that needs to be distinguished from pulmonary emboli. Two theories for the etiology of fat embolus during liposuction have been proposed. The first theory is a mechanical blockage of pulmonary capillaries by fat globules that have entered ruptured vessels. The second proposal, termed *fat embolism syndrome*, describes an inflammatory and biochemical etiology wherein circulating free fatty acids in the pulmonary vasculature damages endothelial cells and pneumocytes. The classic symptoms of fat embolism syndrome include respiratory distress, cerebral dysfunction, and petechial rash.^6^

Immediately after the operation, the fluid and electrolyte balance of this patient should be assessed, and replacement fluids should be administered. Length of postoperative monitoring is determined by the patient's health conditions and amount of aspirate removed. Compression garments are used for as long as 6 weeks to decrease edema.^3^ Narcotics are typically required for the first 48 to 72 hours after the operation for pain control, and then most patients are able to transition to nonsteroidal anti-inflammatory drugs. Assessment of wound healing and potential contour deformities should occur at regular follow-ups. The timeline for recovery is sometimes as little as 1 to 7 days depending on the areas where liposuction was performed. Final results are usually arrived at asymptomatically over 3 to 6 months. Skin contraction is not always uniform and can be expected to be suboptimal in the massive weight loss patient, older patient, and postmenopausal female patient. Laser liposuction, which works through a combination of photoacoustic ablation of adipocytes and selective photothermolysis of fibrous septae, may have a role in facilitating skin tightening in patients via several mechanisms. Some of the mechanisms proposed include the stimulation of neocollagen synthesis and elastin fiber remodeling.^7^ Deformities may be corrected with repeat liposuction or fat grafting.^8^ Our patient underwent touch-up liposuction of the flanks 6 months after the initial operation. Images of the final results are shown later.

## Figures and Tables

**Figure:**